# The mediator role of the feeling of personal unaccomplishment in the effect of unemployment anxiety on depression: a research on business faculty students in Türkiye

**DOI:** 10.3389/fpubh.2024.1421137

**Published:** 2024-08-12

**Authors:** Ibrahim Güran Yumusak

**Affiliations:** Department of International Trade and Finance, İstanbul Sabahattin Zaim University, Istanbul, Türkiye

**Keywords:** unemployment anxiety, depressioni, feeling of personal unaccomplishment, university students, Türkiye

## Abstract

**Background:**

This study aims to determine how the unemployment anxiety of university students affects their depression level. It also examines the mediating role of the feeling of personal unaccomplishment between these effects.

**Methods:**

The data was analyzed using the PROCESS method. The research was conducted on 843 students attending different public or foundation (private) universities in Türkiye. Random sampling was used to select the participants.

**Results:**

As a result of the analysis, it was determined that there is a positive, significant, moderate (*R* = 0.509, *p* < 0.01) relationship between unemployment anxiety and depression and a positive, significant and weak relationship between unemployment anxiety and feeling of personal unaccomplishment (*R* = 0.102, *p* < 0.01), there is a positive, significant, and weak (*R* = 0.184, *p* < 0.01) relationship between feeling of personal unaccomplishment and depression. Unemployment anxiety of university students had significant and positive effects on their depression level and significant and positive effects on feelings of personal unaccomplishment of students.

**Conclusion:**

As a result of the mediating variable analysis, it was determined that the feeling of personal unaccomplishment has a partial mediator role in the relationship between unemployment anxiety and depression.

## 1 Introduction

Depression is one of the most common mental disorders in the world (1). It is accepted as the most common disorder in the general population (2). Today, approximately 300 million people from various age groups are exposed to the different biological, physiological and social effects of this disease (1, 3). It has become very common, especially in recent years. Today, approximately 13% of people deal with varying degrees of depression (4). Approximately 3–4% of people appear to have major depression (5). The prevalence of youth depression has risen during the last ten years, surpassing 11% (6). Nowadays, Different biological, psychological, and social factors cause depression and depression paves the way for the development of many diseases such as epilepsy, diabetes, different cancer, obesity and so on (3). Unemployment anxiety is one of these factors that contributes to mental health issues, particularly depression (7).

Unemployment is a very important phenomenon that creates anxiety in people’s lives (8). Although the average global unemployment rate has fluctuated between 5 and 6% in the last 20 years, the unemployment rate, especially the youth unemployment rate, has increased rapidly in many developed countries, especially in the European Union such as Spain, France and Italy (9). A study conducted by the World Bank in 2005 determined that the biggest problem of university students is unemployment, and 38.5% of university graduates between the ages of 20–24 are looking for a job (10). Unemployment, especially youth unemployment, is also an important problem for Türkiye. Although in Türkiye, the unemployment rate for 15–24-year-olds is 16.6 percent for 2024 (11).

Unemployment creates anxiety in people, and this anxiety directly affects human life and human health. Even after this unemployment situation disappears, many people have to deal with this anxiety for a long time (12, 13). The high rate of youth unemployment causes university students, who are assumed to find employment more quickly, to be left alone with unemployment anxieties throughout their student life. According to 2022 data, while the average time to find a first job for undergraduate graduates was 13.6 months in 2021, this period increased to 13.9 months in 2022 in Türkiye. This situation has been observed mainly in recent years among students who will graduate from business and management sciences or similar faculties. While the average job search period for Economics graduates is 19 months, this was found to be 18.4 months for Business Administration graduates (11). The unemployment problem, which causes significant anxiety for young people, is not specific to Türkiye. Young people in many world countries, even in developed countries, face this problem. The increase in anxiety among university students causes the emergence of many physiological problems, such as depression, mental and heart diseases and so on (14). Therefore, it is very important to determine the factors that cause students’ anxiety and the ways and methods to reduce these anxieties.

It is possible to find many studies in the literature on the relationship between unemployment anxiety and depression (14–17). However, these studies contain some shortcomings in some points. First, most of the studies did not examine the relationships between unemployment anxiety and depression on university students in general, but took the sample group from all unemployed people. Only limited number of recent studies examining the relationships between these two variables from the perspective of university students (15, 18). Nowadays, youth unemployment has become much more common in many parts of the world. That’s why there is a need for studies, especially on young people. Second, none of these studies examined the relationship between unemployment anxiety and depression, especially from the perspective of students attending business faculty. It is important to conduct such studies on university students with more limited sample groups in order to better understand the relationships between variables and to determine the relationships between variables more accurately. It is especially important to examine the relationship between unemployment anxiety and depression among business faculty students who are assumed to have more unemployment anxiety. Finally, there are a limited number of studies in the literature examining the effects of unemployment anxiety on the feeling of personal unaccomplishmet (19). There is no research examining the mediating role of feeling of personal unaccomplishment in the relationship between unemployment anxiety and depression.

This study aims to determine the level of unemployment anxiety among business faculty students and the effects of this anxiety on depression levels. In addition, the study also aimed to determine the mediating role of the feeling of personal unaccomplishment in the effect of unemployment anxiety on depression. Within the scope of the research, firstly, students’ unemployment anxiety levels were determined by collecting data. Then, it was examined whether the unemployment anxiety of the students of the business faculty created a feeling of unaccomplishment in them, and if this situation created a feeling of unaccomplishment, whether this situation led to the development of depression among the students.

## 2 Conceptual framework

### 2.1 Unemployment anxiety

Job is an action in which an individual earns income by using his/her labor to meet his/her needs and make his/her life more meaningful. However, the individual may only sometimes have access to a job. Unemployment, in its broadest definition, is when an individual searches for a job and is ready to work for the determined wage but cannot find a job. The job concept not only meets the individual’s physical needs but also impacts the psychological dimension, such as self-worth, social life, and future expectations. A person needs financial resources to meet basic needs such as food, drink, shelter, and security. In addition, a job appears necessary for self-esteem, self-efficacy, self-perception, self-realization, and social life (20). Unemployment is an important economic and psychological problem, as individuals stay away from it.

Although there has been no significant change in average unemployment rates in the last 20 years, it is seen that especially youth unemployment rates have started to increase in the world (9). Different factors influence the emergence of unemployment, depending on its type. There are also reasons for the increase in unemployment among young people. The lack of work experience of young people causes them to be less preferred by employers. In addition, the fact that young people with higher education are overqualified for some starting jobs and experiencing skill mismatch are among the reasons for youth unemployment. In this case, technology also plays an important role (21).

The increase in youth unemployment causes anxiety among university students who are not yet looking for a regular job but will look for a job in the future. University students frequently experience unemployment anxiety, which has been linked to detrimental impacts on their mental, emotional, and physical well-being (22). Many studies have been conducted on young people’s unemployment anxiety, and as a result of these studies, it has been determined that unemployment anxiety creates many physiological and psychological negative effects such as disruption of sleep patterns, depression, feeling unwell, obesity, feeling of failure, and inability to establish social relationships (23–25).

### 2.2 Depression

Etymologically, depression, which takes its root from the Latin word “dēpressus,” meaning “to be low, to suppress,” has been known as the most common mood problem among psychological disorders in recent years. The word depression is an emotional experience that includes feelings of grief, such as collapse, feeling sad, and decreased functional and vital activity (26). Depression generally represents a depressed mood. This depressed mood is accompanied by many different symptoms, such as pessimistic thinking, social isolation, lack of energy, lack of attention and concentration, sleep problems, and memory problems, and it prevents the individual from continuing his daily life (27). Depression is accompanied by many different symptoms in its clinical course and negatively affects the life of the individual. Goodwin et al. (28) stated in their studies that the clinical course of depression may be resistant to time and may continue to exist effectively in the following years of the individual’s life.

Specific criteria must be met for the development of depression disorder. The diagnostic criteria for depression in the Diagnostic and Statistical Manual of Mental Disorders (DSM), which was developed by the American Psychological Association (APA) and determines the most current and comprehensive criteria for mental disorders, are as follows: depressed mood and loss of interest lasting for 2 weeks; increase or decrease in desire to eat; sleepiness or insomnia problems; decrease in energy, slowing of behavior; feelings of worthlessness and guilt; focus and attention problems; thoughts of self-harm and suicide (29).

The causes of depression, which is characterized by different signs and symptoms, also vary. In this context, in addition to biological explanations, there are etiologies of depression developed in line with the views of psychological schools (30).

Treatment of depression, which has significant effects on physical health as well as mental health, is considered necessary to ensure and maintain well-being. In this context, it is also important to raise awareness about the antecedents and consequences of depression.

There are many studies in the literature explaining the antecedents and consequences of depression. In these studies, many factors such as stress, anxiety, physical problems, health problems and social problems are the cause of depression; it has been found that depression causes many problems such as physical and psychological health problems, obesity, loss of self-esteem and even suicide (31–35).

### 2.3 Unemployment anxiety and depression

Unemployment anxiety can affect an individual’s level of psychological resilience, creating a vulnerable environment for depression. Amiri (2) reported in their study that because depression symptoms and depressive disorder are more common among the unemployed, unemployment may have a detrimental effect on their mental health.

In a study conducted by Choi and Lee (36), a positive relationship was found between students’ physical and mental health and unemployment stress and a negative relationship between self-esteem and unemployment stress. Students have been found to experience more stress when they are female, in higher grades, have low satisfaction with school life, have no free time, are inadequately prepared for employment, and have no one with whom they can share their feelings about unemployment.

Mokona et al. (18) stated that depression is associated with anxiety caused by unemployment in individuals. The results of their study indicate that 30.9% of young adults without jobs had depression overall. Thirty six percent of the study participants with depression had moderate depression, 7.3% had severe depression. The anxiety that young people will be unemployed after Achdut and Refaeli (37) state that COVID-19 causes unemployement anxiety among youth people and this anxiety causes depression among them.

One of the important factors affecting depression is anxiety about the future. Therefore, students’ anxiety about the future may affect their depression levels. For example, in the research conducted by Dağtekin et al. (38), it was found that the depression levels of students who had professional future anxiety were higher than those with low future anxiety.

In their study, Chowdhury et al. (39) reported the effects of the COVID-19 pandemic on unemployment anxiety and depression in young people. In the study, young people’s anxiety about unemployment increased significantly due to the pandemic. The result of their study indicate that 80.2% university students had mild to severe depression and 77.3% reported having low to moderately perceived unemployement stress.

Unemployment is associated with significantly greater rates of depression and anxiety and constitutes a significant public health problem. Arena et al. (40) published 33 articles on mental health-focused interventions for the unemployed were examined. This research aims to present the most comprehensive synthesis and first meta-analysis to date of controlled intervention studies aiming to improve depression and anxiety outcomes during unemployment. According to the findings, although both preventive and treatment interventions are generally effective among those experiencing unemployment, treatment interventions contribute more to reducing anxiety and depression symptoms.

### 2.4 Feeling of personal unaccomplishment

Burnout consists of three sub-dimensions: emotional exhaustion, depersonalization, and a feeling of personal unaccomplishment, and manifests itself with some emotional, behavioral, and physical symptoms in individuals. Some of these symptoms are the purely psychological pressure variable and feeling under pressure and the loss of one’s emotional resources, negative and insensitive responses to people receiving service from a person, decreased personal success in assuming personal responsibilities, and negative evaluations of work (41).

Personal accomplishment is defined as “a sense of competence and successful accomplishment in one’s work with people” (42). The feeling of personal unaccomplishment can be expressed as a person’s negative evaluation of himself/herself and the tendency to fail (43). According to Bandura’s efficacy theory (44), personal success is associated with mastery experience, which constitutes the most critical source of self-efficacy. When people do a task, the feeling of accomplishment gives them confidence and reassurance that they can perform similar tasks successfully. Conversely, if people fail at a task, their self-efficacy and sense of accomplishment weaken (45).

The research conducted by Iacovides et al. (46) on nurses revealed that there was a weak but positive significant relationship between burnout and depression.

In a study conducted by Tanrıverdi and Sarıhan (47), it was determined that there were statistically significant relationships between work engagement, burnout, and depression levels of healthcare professionals. It has been concluded that as healthcare professionals’ level of burnout increases, their depression levels increases too.

Mousavi et al. (19) determined the relationship between burnout dimensions and psychological symptoms (depression, anxiety, and stress) in nurses; it was found that as the sense of personal success increases, the level of mental health also increases. In addition, it is seen that stress-related problems in the workplace can affect the mental health of nurses, causing a feeling of helplessness and depression. Constant stress and anxiety disrupt physical and mental balance and can lead to problems such as leaving work, frequent absences, loss of energy, and decreased work efficiency.

This study was conducted to determine how unemployment anxiety of university students affects their depression level and whether the feeling of personal unaccomplishment plays a mediating role in this interaction. The hypotheses to be used in this study are formed as follows:

*H*1: Unemployment anxiety of students is positively associated with their depression level.

*H*2: The feeling of personal unaccomplishment of students mediates the interaction between their unemployment anxiety and depression level.

## 3 Methods

### 3.1 Sampling

The data obtained within the scope of the research was collected using a survey method. It was employed random sampling, which is a probabilistic method of sampling. The online questionnaire was delivered to all participants via e-mail. The online questionnaire was made available on the Google Forms. An ethics committee decision was taken before the study. Additionally, consent to participate in the study was obtained from all participants. There were no rewards, monetary or otherwise, offered for participating in this research.

A sample of 843 students attending different public or foundation (private) universities in Türkiye was collected. The sample primarily consisted of men (63.0%) aged 19–26 (mean = 22.6; standard deviation = 2.65). A majority of participants are single (96.7%). Most participants described themselves as middle-income level (73.3%). A majority of participants attend public universities (84.9%). A significant portion of the participants receive their education in Turkish (78.2%).

### 3.2 Measures

The questionnaire consists of four parts. The first part aims to determine the demographic characteristics of the participants. The second part includes the unemployment anxiety scale, the third part includes the feeling of personal unaccomplishment scale, and the last part includes the depression scale.

Unemployment anxiety for university students scale was developed by Demir (48). This scale consists of five subgroups and 21 items. Item examples include “Living with constant anxiety about finding a job disrupting affects my psychology” and “I think that I will not be able to find a suitable job because of my gender.” Each item was assessed by a five-point scale ranging from 1 (“totally disagree”) to 5 (“totally agree”). Cronbach alpha score was found to be 0.907.

The feeling of personal unaccompalishment scale is a dimension of the burnout scale developed by Maslach et al. (49). The scale consists of 8 items. Item examples include “I accomplish many worthwhile things in the job,” “In my job I handle emotional problems very calmly.” Each item was assessed by a five-point scale ranging from 1 (“never “) to 5 (“every day”). Cronbach alpha score was found to be 0.814.

Depression levels among university students were evaluated utilizing the Beck Depression Inventory (BDI), which is widely recognized as the primary self-assessment tool for detecting depression (50). The BDI comprises 21 multiple-choice questions designed to gauge the subject’s emotional, cognitive, and physical symptoms. Each question offers four response options, ranging from 0 to 3, with higher scores indicating more pronounced depressive symptoms. It is considered a reliable measure for assessing depression in individuals aged 13 and older. Responses are rated on a 4-point Likert scale, ranging from “Not at all” (0) to “Severely” (3). The total score, derived by summing all items, ranges from 0 to 63. BDI scores were then classified into four categories: minimal or absent depression (0–13), mild depression (14–19), moderate depression (20–28), and severe depression (29–63). Participants scoring in the moderate to severe range were identified as experiencing depression. Cronbach alpha score was found to be 0.890.

The scales used in the research are given in Supplementary Appendix.

### 3.3 Confirmatory factor analyses

Following the parametric test and the correlation results, it was possible to understand the relationships’ patterns among all the constructs. Before hypothesis testing, structural models were tested with the AMOS 25.0 program. The mediated model provided an acceptable fit [χ^2^/df = 3.839; CFI = 0.903; AGFI = 0.832; GFI = 0.862; RMSEA = 0.058]. Structural models were tested with the AMOS 25.0 program. The mediated model provided an acceptable fit [χ^2^/df = 3.839; CFI = 0.903; AGFI = 0.832; GFI = 0.862; RMSEA = 0.058]. A structural model was employed to mitigate common method variance and validate discriminant validity (51). As a result of the analysis, it was indicated that the factor structures of the research variables aligned with the conceptual model (the results presented in Table 1).

**Table 1:** Descriptive statistics and correlation matrix.

	Mean	SD	Skewness	Kurtosis	CR	AVE	MSV	Max R(H)	1	2	3	4	5
Gender	1.37	0.48							1				
Age	22.6	2.65							0.169**	1			
Class	3.03	1.38							0.061	0.580**	1		
Personal unaccompalishment	2.47	0.65	0.487	0.644	0.82	0.515	0.060	0.892	−0.001	−0.034	0.024	1	
Unemployment anxiety	3.71	0.67	−0.604	0.261	0.91	0.571	0.320	0.932	−0.180**	0.113**	0.183**	0.102**	1
Depression level	18.57	10.60	0.742	0.355	0.87	0.627	0.320	0.952	−0.050	0.046	0.090*	0.184**	0.509**

SD, Standard deviation; CR, composite reliability; AVE, average variance extracted; MSV, maximum shared variance; MaxR(H), maximum reliability. **p < 0.01; *p < 0.05.

### 3.4 Descriptive analysis

In this part, basic descriptive analysis, the independent sample *t*-test, the one-way ANOVA test, and correlation analysis were analyzed by the SPSS 25 program.

The mean values showed that the unemployment anxiety of students is at a moderate level (M = 3.71; SD = 0.67). The mean values obtained for personal unaccompalishment (M = 2.47; SD =0.065) and for depression (M = 18.57; SD = 10.60) suggested that students feel a low level of personal unaccompalishment and a moderate level of depression. As a result of the analysis, it is seen that 36.1% of the students are in moderate depression, and 16.4% are in severe depression. It is observed that more than 50% of the participating students are depressed (the results presented in Table 1). It is seen that the depression experienced among university students in Turkiye is higher than in other countries, especially countries with high levels of economic prosperity (52–54).

According to correlation analysis (Table 1), it was indicated that personal unaccompalishment relates positively with unemployment anxiety (*r* = 0.102, *p* < 0.01) and positively with depression (*r* = 0.184, *p* < 0.01). In addition, unemployment anxiety relates positively with depression (*r* = 0.509, *p* < 0.01).

A significant difference was found between the unemployment anxiety among female students is higher than male students (*t* = 5.31, *p* < 0.00). It was also found that the unemployment anxiety and depression levels among foundation (private) university students are higher than public university students (*t* = 3.61, *p* < 0.00; *t* = 8.00, *p* < 0.00). The one-way ANOVA test results determined that as the age of students increases, unemployment anxiety increases too (*F* = 3.62, *p* < 0.01). It is also observed that students experience less unemployment anxiety and depression as their families’ financial situation improves (*F* = 36.43, *p* < 0.01; *F* = 20.03, *p* < 0.01).

### 3.5 Hypothesis testing

In order to evaluate our proposed model, a regression-based path analysis was conducted employing PROCESS v.4.2 macro in SPSS IBM Statistics 25.0 software. This software enables the calculation and examination of interactions as well as the conditional indirect impacts within moderated mediation models (55). The model utilized for executing the PROCESS macro was Model 4. To test the mediation hypothesis, 5,000 bootstrap samples were employed, utilizing a 95% bias-corrected bootstrap confidence interval for all indirect effects.

Hypothesis 1 (H1) proposed that the unemployment anxiety of students would be positively associated with their depression level. The results revealed that the unemployment anxiety of students had a total effect on their depression level, *B* = 7.96, SE = 0.46, *t* = 17.152, *p* < 0.001, H1 was supported (Table 2).

**Table 2:** Results of mediation analysis (hypotheses 1 and 2).

Steps		*B*	*SE*	*t*	*p*-value
Direct and total effects *R*^2^ = 0.27, *p* < 0.001					
Depression level regressed on unemployment anxiety (H1)		7.96	0.46	17.15	<0.001
Depression level regressed on unemployment anxiety, controlling for personal unaccomplishment (H2)		7.75	0.46	16.80	<0.001
	Unstandardized value	*SE*	LL 95% CI	UL 95% CI	
Bootstrap results for indirect effect					
Effect	0.21	0.09	0.053	0.424	<0.001

Listwise *N* = 843. LL, lower limit; CI, confidence interval; UP, upper limit. Bootstrap sample size = 5,000. All predictor variables were mean-centered.

Hypotheses 2 (H2) proposed that the feeling of personal unaccompalishment of students would mediate the relationship between their unemployment anxiety and depression level. It was observed that a significant indirect effect of unemployment anxiety on depression level through the feeling of personal unaccompalishment, indirect effect = 0.21, *p* < 0.001, 95% CI: 0.054–0.424. The direct effect of unemployment anxiety remained significant after controlling for the feeling of personal unaccompalishment, *B* = 7.75, SE = 0.46, *t* = 16.80, *p* < 0.001. It was assumed that feeling of personal unaccompalishment of students has a partial mediation effect between their unemployment anxiety and their depression level, thus supporting H2 (Table 2).

## 4 Conclusion

This study aims to determine how the unemployment anxiety of university students affects their depression level. It also examines the mediating role of the feeling of personal unaccomplishment between these effects. According to the study findings, there is a significant positive relationship between university students’ unemployment anxiety and depression. The results of similar studies previously conducted on different sample groups in the literature support the results of this study (2, 18, 36). According to the study result, it was also determined that the feeling of personal unaccomplishment has mediator role in the relationship between unemployment anxiety and depression. In other words, university students’ unemployment anxiety creates a feeling of personal unaccomplishment. This feeling drags the student into depression.

## 5 Implication and suggestions

According to these study findings, reducing students’ unemployment anxiety is necessary by implementing employment and education policies. Although the permanent solution to the problem is to prevent unemployment among the educated young population, it is impossible to eliminate it. For this reason, it is necessary to develop practices to facilitate the transition of educated young people to the labor market. While the most important of these practices is the expansion of internship opportunities, there is also a need to create courses and units to help students plan their careers in the first years of university. There is also a need to strengthen university-industry cooperation studies and periodically update course curricula, considering the changes in the labor market. When determining the new departments and their quotas, the labor market demands and employment opportunities must also be considered. In addition to this, decreasing the demand for higher education by increasing the interest in vocational high schools can contribute to the normalization of Turkey’s education pyramid. In this way, it may be possible for students to focus on professional fields that require more intermediate staff. Because in Turkey, approximately 1/4 of the students in secondary education are in vocational education and this rate is quite low.

Families bear the costs of university education with the thought that their children will have better job opportunities in society. However, unemployment among young people who are about to graduate from university reinforces their idea of being a burden on the family. This situation increases domestic problems, and prolonging the process also brings psychological problems. For this reason, there is a need to include this in family and social service policies in preventing youth unemployment. Policymakers need to increase rehabilitation efforts to alleviate the psychological negative effects of unemployment on young people. Although there are incentives to increase youth employment in Turkey, additional measures must be taken. In this process, job search allowance or similar measures may be taken to facilitate the employment of young people.

## 6 Limitations

Sampling method, relatively small sample size, and time can be accepted as limitations for this study. Future research should reconfirm this study results by conducting new sampling method and large sample size. The study’s results cannot be generalized to all students and are limited only to the universities where the study was applied. Likewise, it is recommended that the study be carried out with more universities and university students in order to generalize the study to all university students.

## Data availability statement

The raw data supporting the conclusions of this article will be made available by the authors, without undue reservation.

## Ethics statement

The studies involving humans were approved by the İstanbul Sabahattin Zaim University Ethics Commission with the number 2023/04 and the number E81 20292139-050.01.04-54479 on 03.05.2023. The studies were conducted in accordance with the local legislation and institutional requirements. The participants provided their written informed consent to participate in this study.

## Author contributions

IY: Conceptualization, Data curation, Formal analysis, Investigation, Methodology, Supervision, Writing – original draft, Writing – review & editing.
