# Supplementary material for: The mediator role of the feeling of personal unaccomplishment in the effect of unemployment anxiety on depression: a research on business faculty students in Türkiye
Source: Front Public Health. 2024 Aug 12;12:1421137. doi: 10.3389/fpubh.2024.1421137 (PMC11345369; doi:10.3389/fpubh.2024.1421137)
Supplement: Supplementary file 1 [file Data_Sheet_1.docx]

**Appendix**

**Beck's Depression Inventory**

This depression inventory can be self-scored.

1.

1. I do not feel sad.
2. I feel sad
3. I am sad all the time and I can't snap out of it.
4. I am so sad and unhappy that I can't stand it.

2.

1. I am not particularly discouraged about the future.
2. I feel discouraged about the future.
3. I feel I have nothing to look forward to.
4. I feel the future is hopeless and that things cannot improve.

3.

1. I do not feel like a failure.
2. I feel I have failed more than the average person.
3. As I look back on my life, all I can see is a lot of failures.
4. I feel I am a complete failure as a person.

4.

1. I get as much satisfaction out of things as I used to.
2. I don't enjoy things the way I used to.
3. I don't get real satisfaction out of anything anymore.
4. I am dissatisfied or bored with everything.

5.

1. I don't feel particularly guilty
2. I feel guilty a good part of the time.
3. I feel quite guilty most of the time.
4. I feel guilty all of the time.

6.

1. I don't feel I am being punished.
2. I feel I may be punished.
3. I expect to be punished.
4. I feel I am being punished.

7.

1. I don't feel disappointed in myself.
2. I am disappointed in myself.
3. I am disgusted with myself.
4. I hate myself.

8.

1. I don't feel I am any worse than anybody else.
2. I am critical of myself for my weaknesses or mistakes.
3. I blame myself all the time for my faults.
4. I blame myself for everything bad that happens.

9.

1. I don't have any thoughts of killing myself.
2. I have thoughts of killing myself, but I would not carry them out.
3. I would like to kill myself.
4. I would kill myself if I had the chance.

10.

1. I don't cry any more than usual.
2. I cry more now than I used to.
3. I cry all the time now.
4. I used to be able to cry, but now I can't cry even though I want to.

11.

1. I am no more irritated by things than I ever was.
2. I am slightly more irritated now than usual.
3. I am quite annoyed or irritated a good deal of the time.
4. I feel irritated all the time.

12.

1. I have not lost interest in other people.
2. I am less interested in other people than I used to be.
3. I have lost most of my interest in other people.
4. I have lost all of my interest in other people.

13.

1. I make decisions about as well as I ever could.
2. I put off making decisions more than I used to.
3. I have greater difficulty in making decisions more than I used to.
4. I can't make decisions at all anymore.

14.

1. I don't feel that I look any worse than I used to.
2. I am worried that I am looking old or unattractive.
3. I feel there are permanent changes in my appearance that make me look unattractive
4. I believe that I look ugly.

15.

1. I can work about as well as before.
2. It takes an extra effort to get started at doing something.
3. I have to push myself very hard to do anything.
4. I can't do any work at all.

16.

1. I can sleep as well as usual.
2. I don't sleep as well as I used to.
3. I wake up 1-2 hours earlier than usual and find it hard to get back to sleep.
4. I wake up several hours earlier than I used to and cannot get back to sleep.

17.

1. I don't get more tired than usual.
2. I get tired more easily than I used to.
3. I get tired from doing almost anything.
4. I am too tired to do anything.

18.

1. My appetite is no worse than usual.
2. My appetite is not as good as it used to be.
3. My appetite is much worse now.
4. I have no appetite at all anymore.

19.

1. I haven't lost much weight, if any, lately.
2. I have lost more than five pounds.
3. I have lost more than ten pounds.
4. I have lost more than fifteen pounds.

20.

1. I am no more worried about my health than usual.
2. I am worried about physical problems like aches, pains, upset stomach, or constipation.
3. I am very worried about physical problems and it's hard to think of much else.
4. I am so worried about my physical problems that I cannot think of anything else.

**The Feeling of Personal Unaccomplishment Scale**

**Scale**

1. Every day
2. A Few Times per Week
3. A Few Times per Month
4. A Few Times per Year
5. Never

**Items**

1. I accomplish many worthwhile things in the school.
2. I feel full of energy.
3. I not easily able to understand what my patients/friends feel.
4. I look after my patients’/friends’ problems very effectively.
5. In my work, I handle emotional problems very calmly.
6. Through my work, I feel that I have a positive influence on people.
7. I am easily able to create a relaxed atmosphere with my patients/friends.
8. I feel refreshed when I have been close to my patients / friends at scho

**Unemployment Anxiety For University Students Scale**

**Scale**

1. Totally disagree
2. Disagree
3. Neither agree nor disagree
4. Agree
5. Totally agree

**Items**

1. Graduates of my department cannot work in the jobs they want increases my anxiety.
2. I am concerned that the number of appointments made to public institutions is far below the number of graduate students.
3. The high unemployment rate in our country increases my anxiety about finding a job.
4. The fact that my department produces too many graduates reduces my job opportunities.
5. I live with the mentality of "I can't find a job among so many graduates."
6. Living with constant anxiety about finding a job is destroying my psychology.
7. The lack of good working conditions in the private sector narrows my business scope.
8. Requiring work experience to work in the private sector worries me about finding the job
9. I think I will not be able to find enough support from the government to start my own business.
10. I think I can't find the job I want without a friend / supporter.
11. Even if I put forth my personal effort, I think I will not be able to find a good job.
12. I think that the state is inadequate in the fight against unemployment.
13. I think I cannot find a suitable job because of my gender.
14. I am worried about not being able to find a job that suits my religious sensitivities.
15. Negative thoughts of people around me towards my department reduce my personal efforts.
16. Not having an internship during school will make it difficult for me to find a job.
17. "I think I could have found a job more easily if I had done a compulsory internship during university years."
18. Giving lessons based on theoretical knowledge rather than practice increases my anxiety about finding a job.
19. I do not worry about finding a job because I think I will easily find the job I want
20. Since I will start my own business, I do not worry about finding a job.
21. As my level of knowledge in my department increases, my anxiety about finding a job decreases day by day.
